# A rare presentation of perforated Meckel diverticulitis in adulthood

**DOI:** 10.1093/jscr/rjaf348

**Published:** 2025-05-30

**Authors:** Alejandra Viera Plasencia, David A Motola

**Affiliations:** Herbert Wertheim College of Medicine, Florida International University, 11200 SW 8th St, Miami, FL 33199, United States; Department of Surgery, Baptist Health South Florida, 6200 SW 73rd Street, South Miami, FL 33143, United States

**Keywords:** Meckel diverticulitis, Meckel diverticulum, right lower quadrant pain, small bowel disease, minimally invasive surgery, robotic-assisted surgery

## Abstract

Meckel diverticulum is the most common congenital anomaly of the gastrointestinal tract, often asymptomatic but capable of causing complications such as obstruction, bleeding, or diverticulitis. We present a 54-year-old male who presented with acute lower abdominal pain, leukocytosis, and imaging findings suggestive of Meckel diverticulitis. Surgery revealed signs of perforation, with final pathology confirming the diagnosis. The patient recovered well, with complete symptom resolution by postoperative day 14. This case emphasizes the importance of recognizing complicated Meckel diverticulitis as a surgical emergency and highlights the benefits of minimally invasive, robotic-assisted surgery in optimizing patient outcomes.

## Introduction

Meckel diverticulum is the most common gastrointestinal congenital anomaly, resulting from incomplete obliteration of the omphalomesenteric duct, which forms a true diverticulum [1]. During fetal development, the omphalomesenteric duct connects the yolk sac to the midgut and obliterates by the seventh week of gestation. Failure of this process leads to Meckel diverticulum. The diverticulum may contain ectopic tissue, which makes it prone to complications like bleeding or infection [2].

Meckel diverticula usually follow the “rule of twos”: present around the age of 2 years, 2:1 male-to-female ratio, contain two ectopic tissues, present in 2% of the population, occur 2 ft from the ileocecal valve, and are 2 in. long [3]. However, not all cases strictly adhere to this rule. Meckel diverticula may contain ectopic tissue, most commonly pancreatic or gastric mucosa, but colonic or hepatobiliary tissue can also be present. Gastric mucosa can cause ulceration and gastrointestinal bleeding [3].

Most cases are asymptomatic, with complication risk declining from 4% at 16 years old to almost 0% at age 86 [4]. In adults, obstructions can occur in up to 40% of symptomatic cases as a result of adhesions or intussusception. Other complications include diverticulitis, ulceration, or perforation [4]. Diverticulitis is often mistaken for appendicitis and is a frequent complication that can lead to perforation if untreated [5]. Gastrointestinal bleeding is more common in the pediatric population, but may also occur in adults due to ulceration from ectopic gastric mucosa. Additionally, the risk of malignancy is higher in Meckel diverticula than in other parts of the small intestine, including carcinoid tumors, adenocarcinoma, and stromal tumors [5]. This case report highlights the treatment of a patient with perforated Meckel diverticulitis with peritonitis.

## Case report

**Figure 1:**
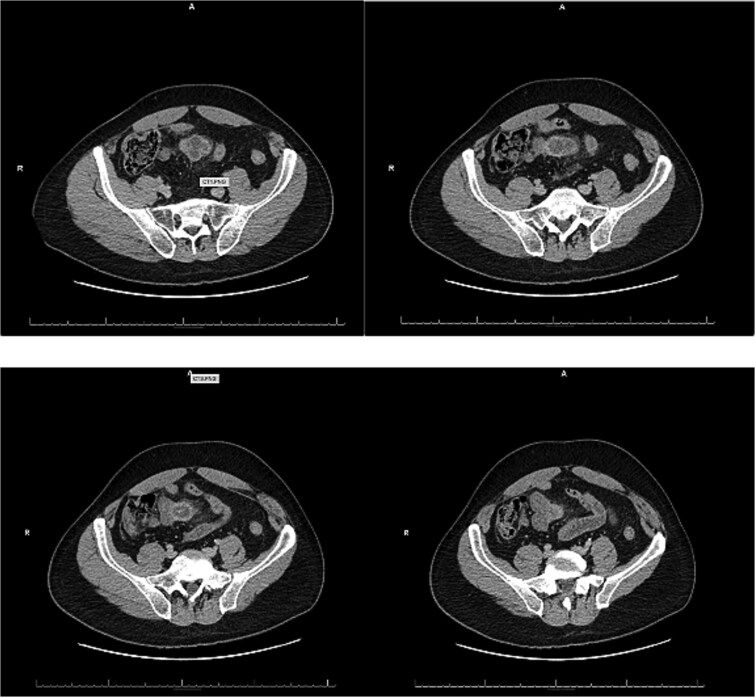
Representative slices of emergency department CT.

A 54-year-old male with a history of hyperlipidemia presented with several hours of worsening lower abdominal pain radiating to the right lower quadrant. He denied nausea, vomiting, diarrhea, or recent illnesses. On examination, he appeared uncomfortable, with a taut, non-distended abdomen, diffuse tenderness, and hypoactive bowel sounds. Laboratory studies revealed leukocytosis (WBC 22.37 K/μL), and an abdominal computed tomography (CT) showed a thick-walled, inflamed, blind-ending tubular structure in the midline pelvis with surrounding fat stranding and small bowel wall thickening, consistent with acute Meckel diverticulitis (Fig. 1). No bowel obstruction or fluid collection was identified. The patient was kept nothing by mouth (NPO), started on IV fluids and broad-spectrum antibiotics, and offered surgical intervention.

**Figure 2:**
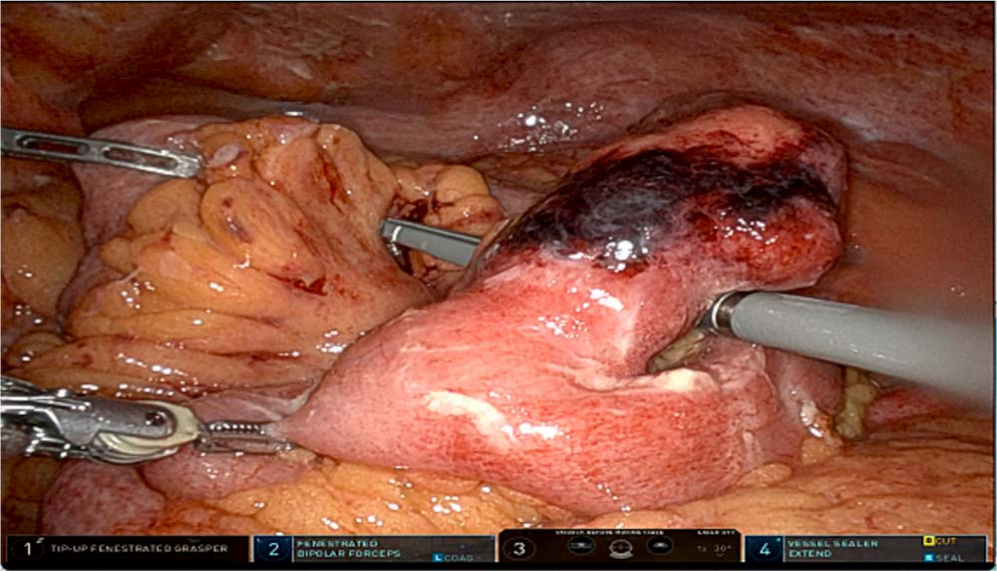
Intraoperative image of Meckel diverticulum.

A robotic-assisted laparoscopic small bowel resection was performed using the da Vinci robotic system (Intuitive, Sunnyvale, CA). Eight mm ports were placed in the left upper quadrant at Palmer point, epigastrium, and left mid-axillary line at the level of the pelvis and a 12 mm port at the left mid-axillary line at the level of the umbilicus. Upon entry, purulent fluid, peritonitis, interloop adhesions, and abscesses were identified (Fig. 2). After adhesiolysis, the inflamed segment was isolated and resected. A stapled, side-to-side isoperistaltic anastomosis was performed. Blood supply was confirmed by near-infrared imaging with indocyanine green. Abscesses were drained, and the specimen was removed. Pathology of the specimen showed a segment of outpouching small intestinal mucosa with marked transmural acute inflammatory infiltrate, stricturing, mucosal denudation, and fibrinopurulent serosal adhesions, consistent with Meckel diverticulum.

Postoperatively, the patient progressed well, transitioned from clear liquids to a full diet, and was discharged on oral antibiotics for 1 week. At his 1-week follow-up, he reported persistent bloating and fatigue but was otherwise stable. By postoperative day 14, he had fully recovered and returned to baseline.

## Discussion

This case highlights the rare presentation of perforated Meckel diverticulitis with peritonitis in an adult patient. Due to the extent of inflammation and sepsis risk, surgical intervention was required. Robotic-assisted resection was chosen for its advantages in reducing surgical complications, post-operative pain, lowering the risk of infection, shortening hospital stay, and allowing for a faster recovery [6]. Advanced technologies, including robotic staplers and near-infrared imaging, enhanced precision in bowel resection and anastomosis, improving clinical outcomes [6].

As previously mentioned, the most common presentation of a symptomatic Meckel diverticulum in adults is with diverticulitis or obstruction, with perforation being far less frequent. According to the literature, in patients with complicated Meckel diverticulum, diverticulitis or small bowel obstruction was observed in ⁓35% of patients, and gastrointestinal bleeding was present in 29% of patients [7]. Additionally, studies suggest that only ⁓4% of symptomatic cases in adults progress to perforation [4], highlighting the rarity of our patient’s presentation.

Patients with Meckel diverticulitis typically present with right lower quadrant pain, fever, and leukocytosis. Additionally, CT imaging reveals a blind-ended pouch with mural thickness, mesenteric inflammation, or air-fluid levels [8]. In our patient, CT findings were suggestive of acute Meckel diverticulitis but did not indicate perforation or abscess formation. However, laparoscopy revealed purulent peritonitis and abscesses, confirming the presence of complicated diverticulitis with probable perforation.

The standard treatment for symptomatic Meckel diverticulum is surgical resection via diverticulectomy, wedge resection, or segmental ileal resection with anastomosis [6]. However, the approach to managing incidentally detected Meckel diverticulum remains controversial. Some studies suggest prophylactic resection of the diverticulum in patients with risk factors such as age younger than 50 years, diverticular length ˃2 cm, and the presence of ectopic tissue within the diverticulum [9]. Other studies recommend that resection should be individualized, given that there is a 1% chance of complications from the prophylactic resection, as compared to a lifelong potential complication of 5% [10].

For small bowel perforation, the preferred approach depends on clinical status. In hemodynamically stable patients with minimal peritoneal contamination, laparoscopic resection, and primary anastomosis are the standard of care [11]. Given our patient’s intraoperative findings of perforation with purulent peritonitis, surgical intervention was necessary. Management includes resection of the perforated small bowel segment with primary anastomosis, followed by a 5-day course of antibiotics covering anaerobes and gram-negative bacteria or other identified organisms [11].

Although the patient experienced mild postoperative bloating and discomfort, his rapid recovery highlights the benefits of early surgical intervention and minimally invasive techniques. This case underscores the importance of recognizing complicated Meckel diverticulitis as a surgical emergency and the advantages of robotic-assisted surgery in improving patient outcomes.

## References

[1] Kuru S, Kismet K. Meckel's diverticulum: clinical features, diagnosis and management. Rev Esp Enferm Dig 2018;110:726–32. 10.17235/reed.2018.5628/2018 30032625

[2] Malik AA, Shams-ul-Bari WKA, Khaja AR. Meckel's diverticulum-revisited Saudi. J Gastroenterol 2010;16:3–7. 10.4103/1319-3767.58760 PMC3023098 20065566

[3] Fusco JC, Achey MA, Upperman JS. Meckel's diverticulum: evaluation and management. Semin Pediatr Surg 2022;31:151142. 10.1016/j.sempedsurg.2022.151142 35305798

[4] Hernández JD, Valencia G, Girón F, et al. Meckel's diverticulum: analysis of 27 cases in an adult population. Front Surg 2023;10:1327545. 10.3389/fsurg.2023.1327545 38179318 PMC10765580

[5] Lequet J, Menahem B, Alves A, et al. Meckel's diverticulum in the adult. J Visc Surg 2017;154:253–9. 10.1016/j.jviscsurg.2017.06.006 28698005

[6] Diana M, Marescaux J. Robotic surgery. Br J Surg 2015;102:e15–28. 10.1002/bjs.9711 25627128

[7] Parvanescu A, Bruzzi M, Voron T, et al. Complicated Meckel's diverticulum: presentation modes in adults. Medicine (Baltimore) 2018;97:e12457. 10.1097/MD.0000000000012457 30235734 PMC6160168

[8] Lindeman RJ, Søreide K. The many faces of Meckel's diverticulum: update on management in incidental and symptomatic patients. Curr Gastroenterol Rep 2020;22:3. 10.1007/s11894-019-0742-1 31930430

[9] Stallion A, Shuck JM. Meckel's diverticulum. In: Holzheimer RG, Mannick JA (eds). Surgical Treatment: Evidence-Based and Problem-Oriented. Munich: Zuckschwerdt, 2001. Available from: https://www.ncbi.nlm.nih.gov/books/NBK6918/21028753

[10] Choi SY, Hong SS, Park HJ, et al. The many faces of Meckel's diverticulum and its complications. J Med Imaging Radiat Oncol 2017;61:225–31. 10.1111/1754-9485.12505 27492813

[11] Coccolini F, Sartelli M, Sawyer R, et al. Source control in emergency general surgery: WSES, GAIS, SIS-E, SIS-A guidelines. World J Emerg Surg 2023;18:41. 10.1186/s13017-023-00509-4 37480129 PMC10362628

